# Chlorhexidine gluconate enhances the remineralization effect of high viscosity glass ionomer cement on dentin carious lesions in vitro

**DOI:** 10.1186/s12903-022-02098-1

**Published:** 2022-03-05

**Authors:** Patcharanun Borompiyasawat, Boonsong Putraphan, Sureerat Luangworakhun, Waleerat Sukarawan, Oranuch Techatharatip

**Affiliations:** 1 Department of Pediatric Dentistry, Faculty of Dentistry, Chulalongkorn University, Bangkok, Thailand; 2 Oral Biology Research Center, Faculty of Dentistry, Chulalongkorn University, 34 Pathumwan, Bangkok, 10330 Thailand; 3 Department of Pediatric Dentistry, Faculty of Dentistry, Chulalongkorn University, 34 Pathumwan, Bangkok, 10330 Thailand

**Keywords:** Chlorhexidine, Glass ionomer cement, Atraumatic restorative treatment, Dental caries, Cavity disinfectant, Primary teeth, Mean mineral density, Micro computed tomography, Remineralization

## Abstract

**Background:**

To compare the mean mineral density (MMD) and examine the remineralization of carious dentin after cavity disinfection with chlorhexidine gluconate (CHX) and restoration with high viscosity glass ionomer cement (H-GIC) in vitro.

**Methods:**

Selective caries removal to leathery dentin was performed in 40 extracted primary molars. The samples were scanned using micro-computed tomography (micro-CT) to determine the MMD baseline and randomly divided into 4 groups (n = 10): Equia™ group, applied dentin conditioner and restored with H-GIC (Equia Forte™), CHX-Equia™ group, disinfected the cavity with 2% CHX before applying dentin conditioner and restored with H-GIC (Equia Forte™), Ketac™ group, restored with H-GIC (Ketac Universal™) and CHX-Ketac™ group, disinfected the cavity with 2% CHX before restored with H-GIC (Ketac Universal™). The samples underwent micro-CT scanning post-restoration and post-pH-cycling to determine their respective MMDs. One sample from each group was randomly selected to analyze by scanning electron microscopy (SEM).

**Results:**

The MMD gain in the 4 groups post-restoration was significantly different between the Equia™ and CHX-Ketac™ groups (oneway ANOVA with Post hoc (Tukey) test, *P* = 0.045). There was a significant difference in MMD gain post-restoration between the Equia™ and CHX-Equia™ groups (Independent t-test, *P* = 0.046). However, the Ketac™ and CHX-Ketac™ group’s MMD were similar. The SEM images revealed that the CHX-Ketac™ group had the smallest dentinal tubule orifices and the thickest intertubular dentin among the groups. However, the CHX-Equia™ group had thicker intertubular dentin than the Equia™ group.

**Conclusion:**

Applying 2% CHX on demineralized dentin enhances the remineralization of the dentin beneath the restoration.

## Background

Dental caries management has evolved into a preventive and restorative strategy known as minimal intervention, which promotes preserving tooth structure and emphasizes maximum tooth function [1–5]. One evidence-based management technique is selective caries removal that is used in deep caries without any signs or symptoms of pulpal degeneration [6]. The concepts of selective caries removal are removing the surrounding axial-wall caries and leaving the pulpal wall caries in the cavity.

Selective caries removal is often used in atraumatic restorative treatment (ART), which is a method to manage deep caries lesions using only hand instruments to reduce trauma to the pulp [7]. ART was developed mainly for treating caries in children living in under-served areas where resources are limited [8]. In addition to reducing pulpal damage, ART results in a reduced pain experience, increased patient cooperation, and is more cost-effective than the conventional treatment [8–10]. Therefore, ART is suitable for pediatric patients who have multiple severe caries, prevention programs, and arresting caries progression. Although the selective caries removal method has a high survival rate, defective restorations, pulpal inflammation, and secondary caries can cause ART failure [11].

There are several ways to improve the success rate of ART. Selecting the appropriate restorative material is an important concern for ART. Glass ionomer cement (GIC) is commonly used in pediatric dentistry, with desirable properties, such as being biocompatible to the tooth or soft tissue, fluoride release, antimicrobial activity, coefficient of material expansion that is similar to tooth expansion, and physio-chemical bonding with tooth structure. The other advantages of glass ionomer are its white color and being more tolerant to moisture compared with resin composite [12]. High viscosity glass ionomer cement (H-GIC) is a material that has been used in ART [13]. ART using H-GIC makes dental treatment easier, faster, and more comfortable than the conventional restorative treatment [14]. A low evidence-based study found that ART using H-GIC demonstrated a higher restoration failure rate in both primary and permanent teeth compared with the conventional treatment [8]. The survival rate in a 2-year follow-up of single surface ART using GIC was high in both primary and permanent posterior teeth, while the multiple surface restorations had a medium survival rate [15]. Although H-GIC was not recommended for multiple surface restoration in primary molars in the past [16], currently a new generation of H-GIC, such as Equia Forte™ (GC Corporation, Tokyo, Japan) and Ketac™ Universal Aplicap™ (3M ESPE Dental Products, St. Paul, USA), are claimed by the manufacturers to be appropriate for restoring cavity class II cavities. The hybrid technology in Equia Forte™ increases flexural strength [17], which prevents material deformation against chewing forces [18].

The hypermineralized zone of the dentin adjacent to a GIC restoration decreases the progression of secondary caries [19, 20]. The hypermineralized zone occurs from the exchange of charged ions between the restorative material and tooth structure [19]. H-GIC contains several minerals that promote a hypermineralized zone in the adjacent dentin [21]. Fluoride and strontium ions from H-GIC can penetrate deep into carious demineralized dentin and induce remineralization [21].

Importantly, antimicrobial agents reduce long-term treatment failure by inhibiting the growth of residual bacteria in deep caries [22]. Antimicrobial agents, such as triclosan, cetylpyridinium chloride, povidone iodine, hydrogen peroxide, sodium hypochlorite, and chlorhexidine gluconate (CHX) are used in different dental products [23–26]. Several studies evaluated using antimicrobial agents as a cavity disinfectant before placing the restoration [27, 28]. CHX is a well-known antimicrobial agent used as a cavity disinfectant in ART [27]. CHX is a broad-spectrum synthetic disinfectant agent that is active against Gram-positive, Gram-negative bacteria and against fungi and viruses [29]. CHX increases the permeability of the bacterial cell membrane, resulting in macromolecules leaking into the cytoplasm and causing cell lysis [29]. CHX is positively charged and eliminates microorganisms by interacting with their negatively charged membrane. CHX reduces *Enterococcus faecalis*, which is difficult to eliminate and known to induce pulpal and periapical inflammation over the long-term, in deep caries lesions [22, 30].

In addition to its antimicrobial effect, several studies found that using CHX with polyacrylic acid increased the GIC bond strength [31–34]. CHX neutralized the dentin surface that was applied with an acid conditioner [33] and also increased the surface energy of the dentin [33]. However, the effect of CHX on the remineralization of the affected dentin after GIC restoration is not clear [35].

Therefore, the aim of this study was to investigate the remineralization effect of CHX used as a cavity disinfectant on dentin carious lesions restored with H-GIC.

## Methods

### Sample size calculation and teeth selection

The study protocols were approved by the Human Research Ethics Committee of the Faculty of Dentistry, Chulalongkorn University (HREC-DCU2020-043), and were approved by the Institutional Biosafety Committee of the Faculty of Dentistry, Chulalongkorn University (DENT CU-IBC 032/2020). The sample size calculation using the G*Power 3.1 program indicated that 6.25 samples were required per group (α = 0.05, β = 0.20). To increase the power of the study, the sample size per group in this study was 10 (40 samples total). The primary molars were collected from the Pediatric Dentistry Department Clinic, Faculty of Dentistry, Chulalongkorn University. The inclusion criteria were extracted primary molars with an occlusal or proximal dentin carious lesion with or without pulpal exposure. If the carious lesion exposed the dental pulp, the exposure size must less than 1 × 1 mm^2^ after selective caries removal. If the carious lesion did not expose the dental pulp, the lesion must invade the dentin based on visual examination. The remaining tooth structure must be more than 1/3 of the crown, and the roots of the teeth must be at least 1 mm long.

### Specimen preparation

The extracted primary molars with carious lesions were stored in 0.9% sodium chloride solution and 10% formalin solution at room temperature at least 2 weeks as previously described [36]. The teeth were cleaned with pumice, rinsed in deionized water, and dried with tissue paper. To prepare a horizontal surface, the cusps of the teeth were cut to a flat occlusal surface with a slow speed cutting machine. The teeth were placed in a prefabricated 18 × 22 × 20 mm^3^ resin mold and attached to the resin mold with dental pink wax. The tooth caries was removed with the ART method using only a spoon excavator, rinsed with water, and dried with sterile cotton pellets. The soft carious tissue in the lesion was removed followed by selective caries removal to leathery dentin. The samples’ dentin mean mineral density (MMD) was measured using micro-CT as baseline.

The teeth were assigned to 4 groups (n = 10) using Permuted block randomization. Equia™ group: the samples were treated with a dentin conditioner (GC Corporation, Tokyo, Japan) for 20 s, rinsed with water, restored with H-GIC (Equia Forte™) and coated with petroleum jelly. CHX-Equia™ group: the sample cavities were applied with 2% CHX liquid for 1 min with a micro-brush according to previous studies [27, 37, 38]. The cavity was treated with a dentin conditioner for 20 s, rinsed with water, restored with H-GIC (Equia Forte™) and coated with petroleum jelly. Ketac™ group: the samples were restored with H-GIC (Ketac Universal Aplicap™). CHX-Ketac™ group: the samples’ cavities were applied with 2% chlorhexidine gluconate liquid for 1 min with a micro-brush. The cavity was restored with H-GIC (Ketac Universal Aplicap™). The samples were stored in sterilized artificial saliva (0.75 gr Potassium chloride, 0.07 gr Magnesium chloride, 0.199 gr Calcium chloride, 0.965 gr, di-Potassium hydrogen phosphate, 0.439 gr Potassium dihydrogen phosphate, 6 gr Sodium carboxymethyl cellulose, 36 gr Sorbital and 2.4 gr Sodium benzoate in a final volume of 1000 ml. The artificial saliva were sterilized in an autoclave at 121 °C, 15 psi for 15 min). The sample were stored at 37 °C for 24 h before micro-CT scanning as the MMD post-restoration.

### pH cycling

The demineralization-remineralization cycling was performed based on Dias et al. [39], as derived from Ten Cate [40]. The samples were immersed in a demineralization solution for 8 h and a remineralization solution for 16 h per day. The samples were stored in individual tubes. The cycle was performed for 14 days at room temperature without stirring. After pH cycling, the samples were soaked in water for 5 min before undergoing micro-CT scanning to determine the post-pH cycling MMD. The solutions used in the pH cycling were prepared by the Biochemistry Department, Faculty of Dentistry, Chulalongkorn University. The demineralization solution (pH 4.8) was composed of 2.2 mM calcium chloride, 2.2 mM sodium phosphate, and 50 mM acetic acid. The remineralization solution (pH 7.0) was composed of 1.5 mM calcium chloride, 0.9 mM sodium phosphate, and 0.15 mM potassium chloride.

### Micro-CT MMD assessment and data analysis

The samples were scanned using micro-CT at baseline, post-restoration, and post-pH cycling. The MMD of each sample was calculated by Micro-CT programs (Micro-CT Ray Version 4.2 and Micro-CT Evaluation Program Version 6.6). The Micro-CT scanning programs were set at a resolution of 1024 × 1024 megapixels compared with hydroxyapatite mineral density 1200 mg/cm^3^, 70 kVp, and 57 μA.

**Fig. 1:**
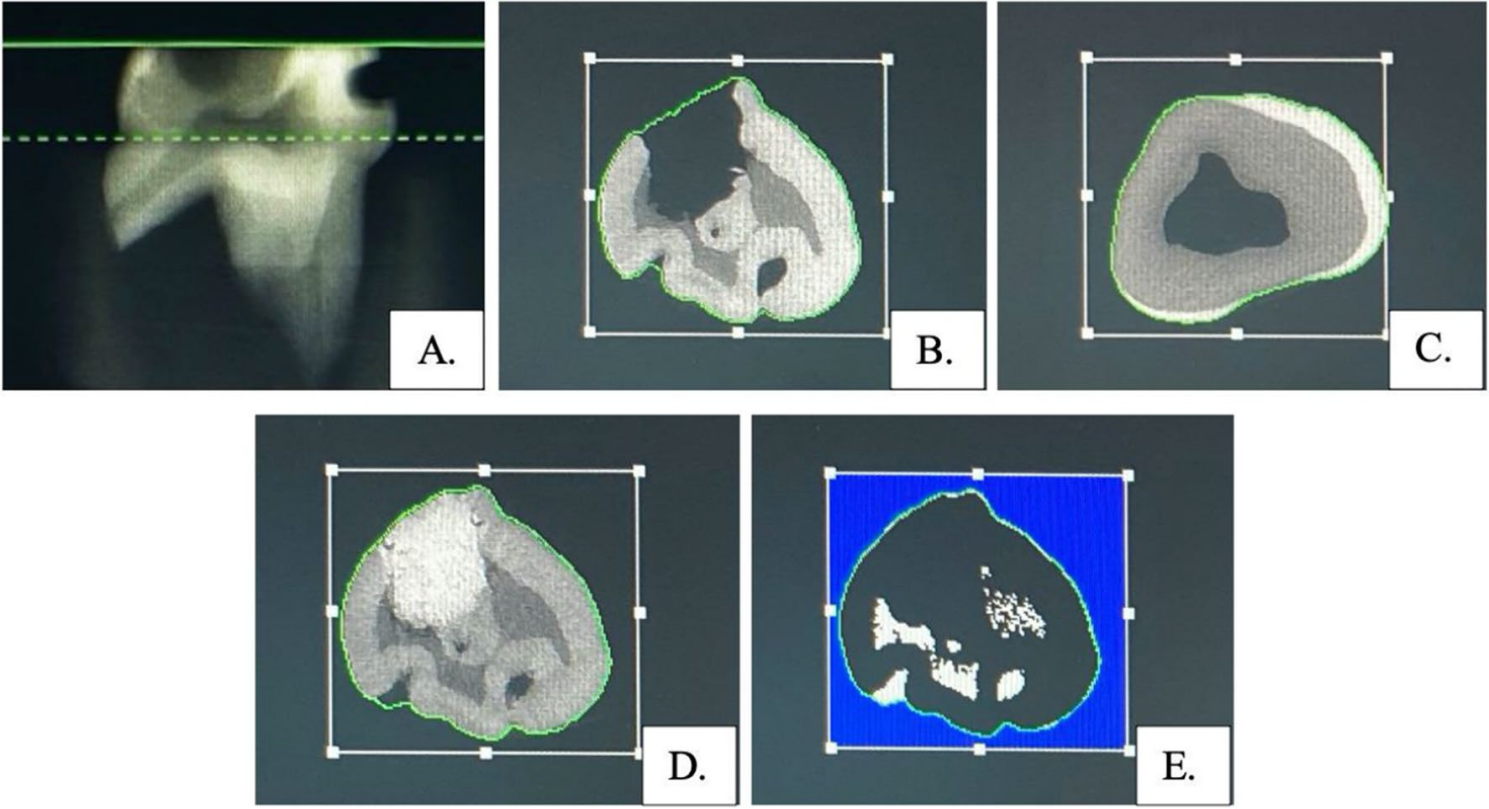
Representative micro-CT scanned images. **A** The micro-CT scanned area. Upper green straight line showed the first occlusal limit which was included the first occlusal slide, lower green dash line showed the lower limit including the roof of pulp chamber. **B** The first slide of the occlusal surface from the baseline sample. **C** The first slide of the roof of pulp chamber from the baseline sample. **D** The after-restoration slide showed the difference contrast between H-GIC and dentin. **E** The preview selecting area of slide with restoration in contrast management, black area showed the excluding area such as restoration and enamel which had high resolution like the restoration and white area showed the including dentin area for mineral density calculation

Before the micro-CT scanning, the image was shown in the program in the 2D sagittal plane. The scanned area was demarcated with green lines. The upper green straight line was the upper scanned limit; the lower green dashed line was the lower scanned limit (Fig. 1A). The scanned area of interest comprised the area from the first slice of the occlusal surface to the first slice of the roof of pulp chamber. The scanning results are presented as slices in the horizontal plane (Fig. 1B, C). The multiple slices of the area of interest were drawn anti-clockwise around the outer surface of the tooth for all tooth surface selection.

The 3D bone morphology analysis program was used to analyze the MMD. The contrast setting for the analysis was determined using the after-restoration samples because the contrast resolution between the H-GIC and dentin in the after-restoration samples was differentiated easier than the dentin alone in the baseline samples (Fig. 1D, E). The contrast resolution was derived from the lowest mean values between 2 examiners who identified the difference in contrast excluding the restoration in all samples. Therefore, the micro-CT contrast value setting was performed at − 1000 for the lower threshold and + 550 for the upper threshold on the micro-CT 3D program. After setting the contrast, the 3D image was constructed from the area of interest slices at baseline, post-restoration, and post-pH cycling.

### SEM preparation

One sample from each group was selected by random number sampling to use in the SEM analysis. The samples in each group were numbered from 1 to 10 and a number from 1 to 10 was randomly selected. The sample from each group with that number was used in the SEM analysis.

The 4 selected samples were cut in the horizontal plane with a slow speed cutting machine and were dried in a critical point dryer (Quarum Model K850). The samples were sputtered with a thin layer of gold and attached to aluminum stubs (JSM-IT300, JEOL, Japan). The surface morphology of the sample was observed using SEM (Quanta 250; FEI Company, Netherlands) at 60× and 5000×. The SEM results represented the morphology of the adjacent dentin beneath the GIC restoration with or without chlorhexidine gluconate treatment on each 5000× magnification SEM micrograph, 3 random tubule diameters were measured by SEM [37].

### Statistical analysis

Descriptive statistics described the mineralization on the dentin carious lesions under the restoration from the SEM image of each group’s sample. The Shapiro–Wilk test and Levene’s test were performed to test the normality and homogeneity of variance of the MMD of carious dentin, respectively. The MMD was compared (1) between baseline/post-restoration or baseline/post-pH cycling in the same group using the paired t-test, (2) between groups using one way ANOVA with Post hoc (Tukey) test. The MMD gain was compared (1) between groups using one way ANOVA with Post hoc (Tukey) test, (2) between the Equia^TM^/CHX-Equia^TM^ groups or Ketac^TM^/CHX-Ketac^TM^ groups using the independent t-test. For all statistical analyses, the tests were performed at the 95% confidence level using SPSS statistic 22.

## Results

### Mean mineral density

**Fig. 2:**
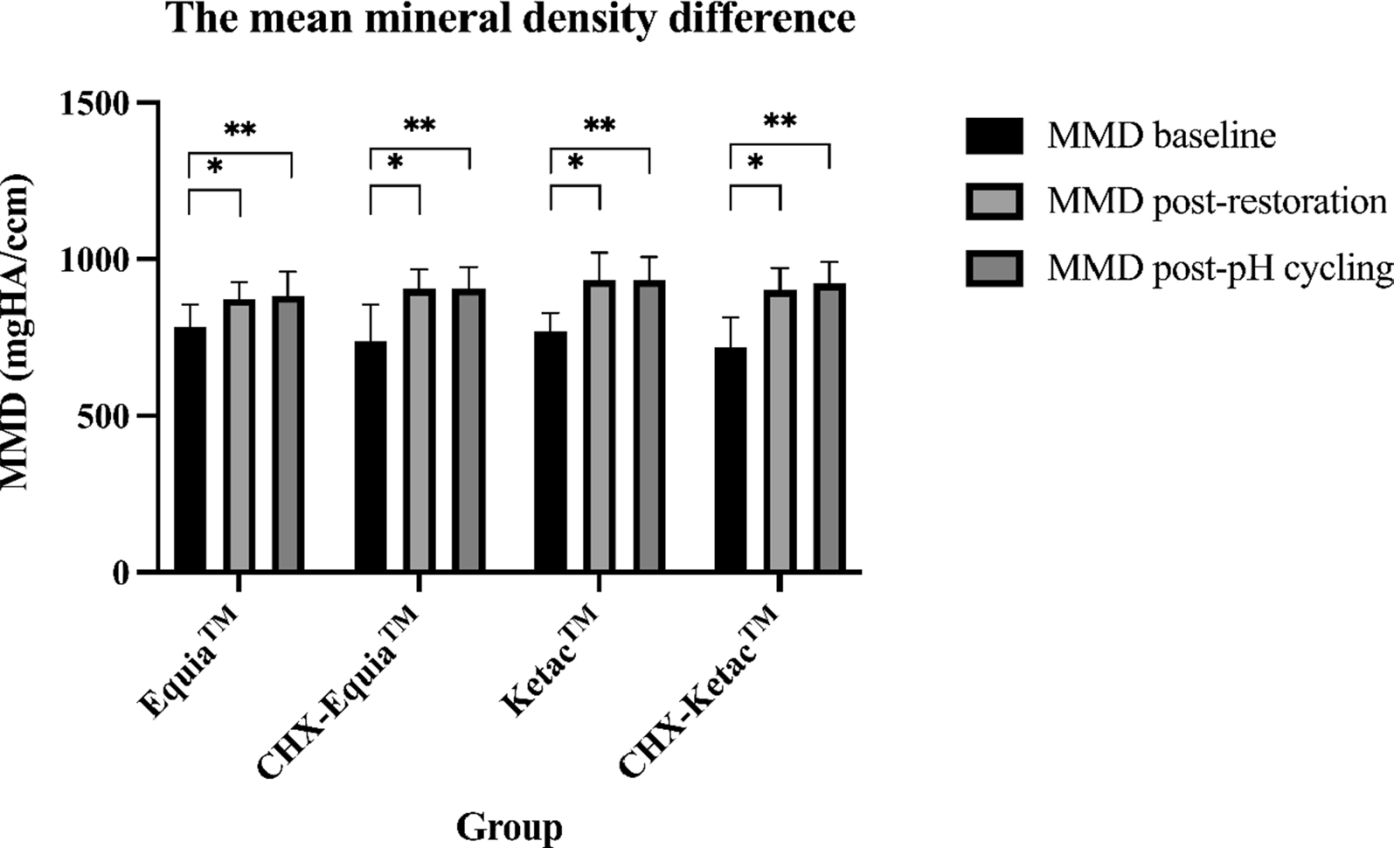
The comparison of mean mineral density of all groups

We determined the MMD at baseline, post-restoration, and post-pH cycling (Table 1). There were no significant differences in MMD at baseline, post-restoration, or post-pH cycling between the groups (one way ANOVA with Post Hoc (Tukey) test, *P* = 0.356, *P* = 0.299, and *P* = 0.419, respectively) (Table 1, Fig. 2). The MMD in all groups (post-restoration and post-pH cycling) were significantly increased compared with baseline (Paired t-test, *P* < 0.001) (Fig. 2).

**Table 1:** The mean mineral density difference between 4 groups

GROUP	A: Equia™ (mgHA/ccm)	B: CHX-Equia™ (mgHA/ccm)	C: Ketac™ (mgHA/ccm)	D: CHX-Ketac™ (mgHA/ccm)	*P*
Mean mineral density baseline	782.74 ± 71.238	735.31 ± 119.479	766.99 ± 59.361	717.10 ± 94.508	0.356
Mean mineral density post-restoration	871.55 ± 54.160	903.60 ± 63.015	932.54 ± 87.099	900.10 ± 71.427	0.299
Mean mineral density post-pH cycling	881.94 ± 77.213	904.52 ± 68.776	933.31 ± 73.769	922.26 ± 68.670	0.419

**Fig. 3:**
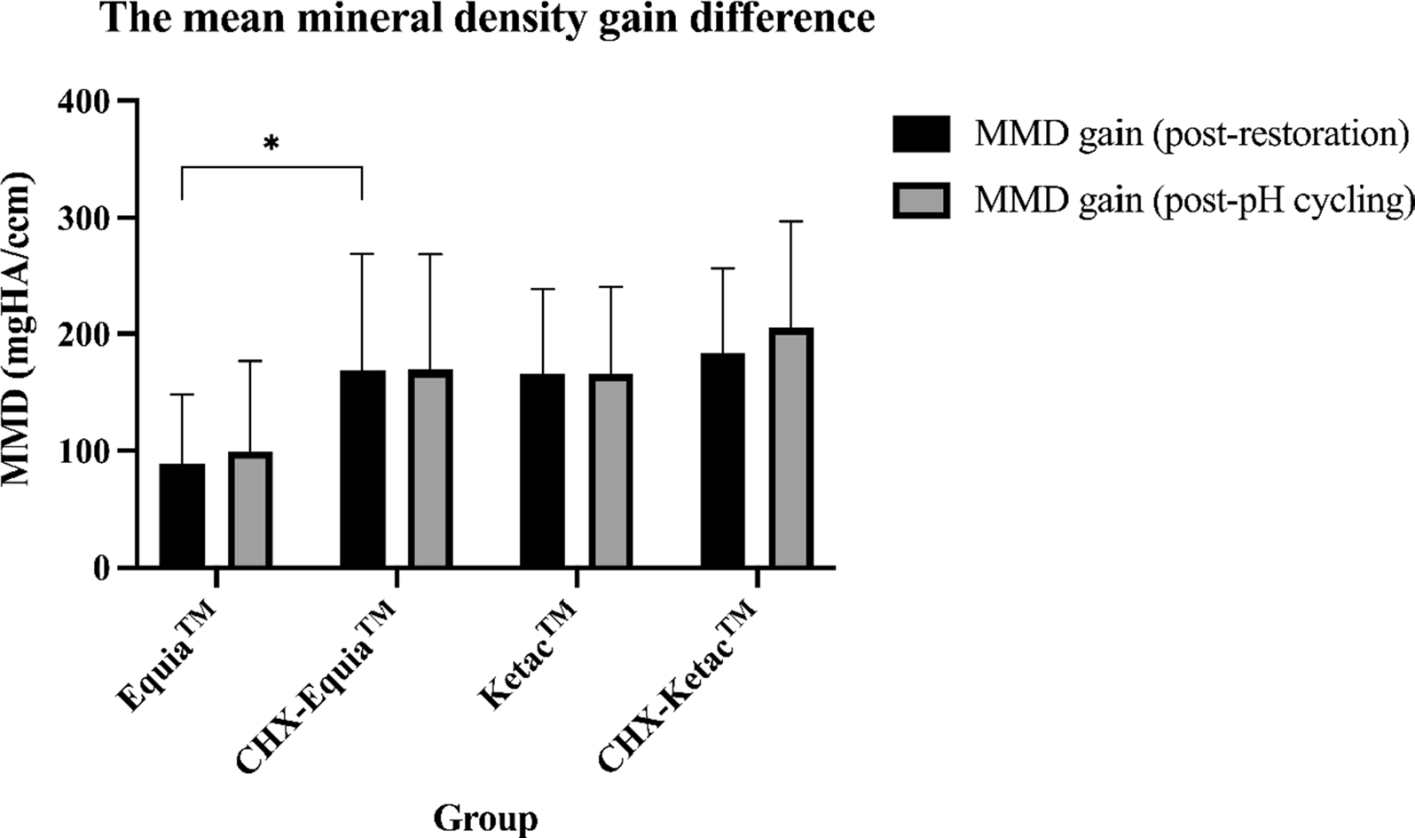
The comparison of mean mineral density gain post-restoration between Equia™/CHX-Equia™ group and Ketac™/CHX-Ketac™ group

The MMD gain is shown in Table 2. The MMD gain post-restoration between the groups was significantly different (One way ANOVA, *P* = 0.045) (Table 2). The Post Hoc (Tukey) test revealed a significant difference between the Equia™ and CHX-Ketac™ groups (*P* = 0.049). In contrast, the MMD gain post-pH cycling between the 4 groups was not significantly different (oneway ANOVA, *P* = 0.065) (Table 2). However, there was a significant difference in MMD gain post-restoration between the Equia™ and CHX-Equia™ groups (Independent t-test*, P* = 0.046) (Fig. 3).

**Table 2:** The mean mineral density gain difference between 4 groups

GROUP	A: Equia™ (mgHA/ccm)	B: CHX-Equia™ (mgHA/ccm)	C: Ketac™ (mgHA/ccm)	D: CHX-Ketac™ (mgHA/ccm)	*P*
Mean mineral density gain (post-restoration)	88.81 ± 59.857	168.29 ± 100.899	165.54 ± 72.366	183.00 ± 73.096	0.045*
Mean mineral density gain (post-pH cycling)	99.20 ± 77.240	169.21 ± 99.199	166.32 ± 74.182	205.15 ± 91.678	0.065

*Comparison the mean mineral density gain post-restoration of 4 groups, significant difference (*P* < 0.05)

### SEM

**Fig. 4:**
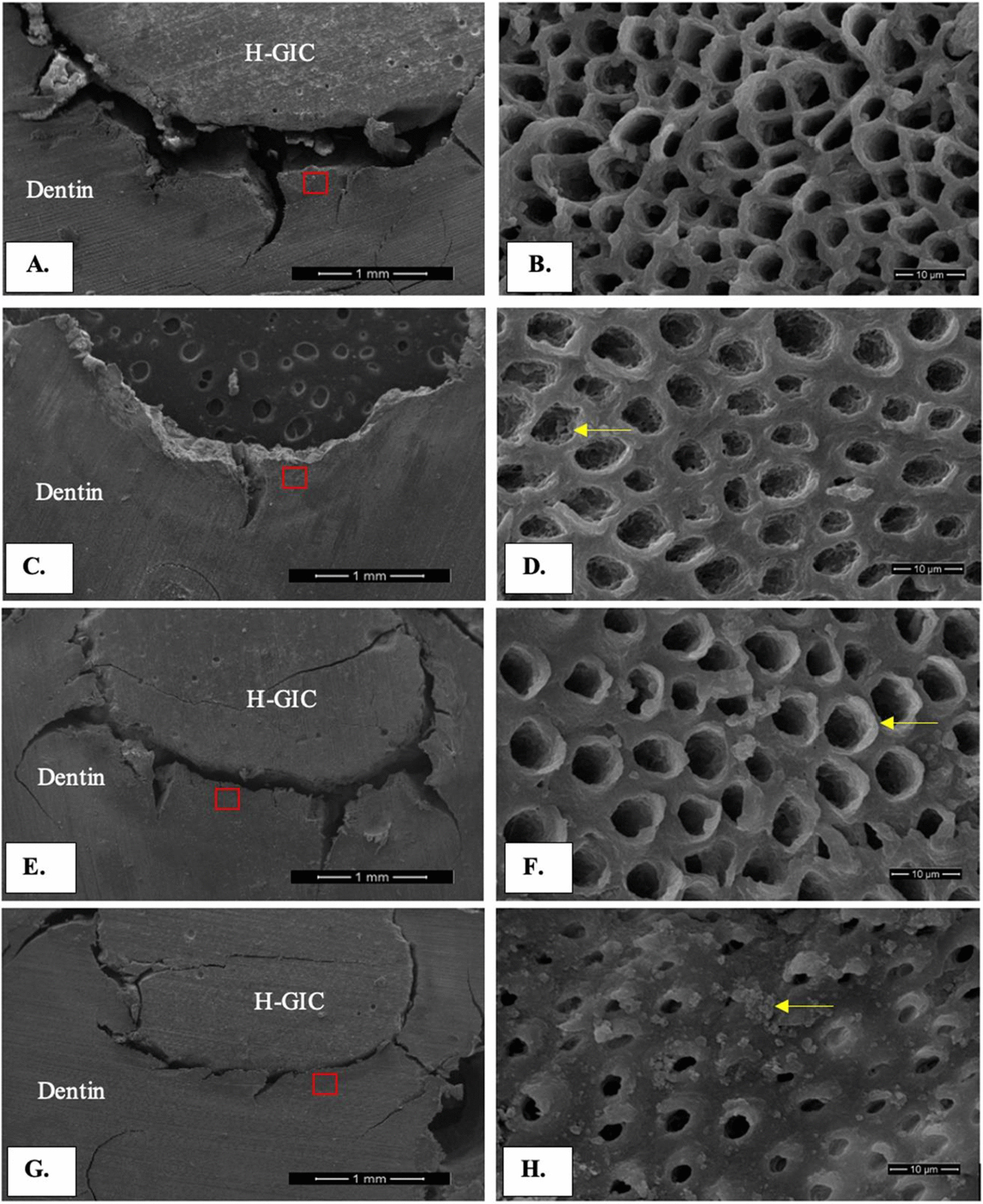
Dentin which contacted H-GIC restoration micrographs of SEM images at 60X magnification **A** Equia™ group, **C** CHX-Equia™ group, **E** Ketac™ group and **G** CHX-Ketac™ group and 5000X magnification **B** Equia™ group, **D** CHX-Equia™ group; the arrow represented inside surface of intratubular dentin, **F** Ketac™ group; the arrow represented peritubular dentin and (H.) CHX-Ketac™ group; the arrow represented particles on the dentin surface

The morphology of the dentin surface in contact with the H-GIC restorations in each group was investigated by SEM at 60× and 5000× magnification (Fig. 4). The H-GIC restoration and adjacent dentin was seen in all groups, except for the CHX-Equia™ group because the restoration was dislodged during specimen preparation (Fig. 4A, C, E, F). The area of the dentinal tubules in the red rectangles are shown at 5000× magnification.

In the Equia™ group, dentin conditioner was applied before restoring with H-GIC (Equia Forte™). The surface of the intertubular dentin appeared eroded with an uneven surface (Fig. 4B). The dentinal tubule orifices were visible, with little if any, peritubular dentin present. Furthermore, the margins of the dentinal tubule orifices were rounded. The dentinal tubules were approximately 5–7 m wide.

In the CHX-Equia™ group, 2% CHX was applied for 1 min followed by the dentin conditioner and restored with H-GIC (Equia Forte™). In contrast to the group treated only with the dentin conditioner, the dentin surface was flat, with wide intertubular dentin present (Fig. 4D). The dentinal tubule orifices were irregularly shaped and were approximately 4–6 µm wide. Peritubular dentin was not always present at the tubule orifice. The inner surface of the peritubular dentin was rough with some particles in the deep part of the dentinal tubules (arrow).

In the Ketac™ group, the cavity was directly restored with H-GIC (Ketac Universal™) without prior treatment. Wide, flat intertubular dentin was observed (Fig. 4F). Deposited material was seen in the intertubular dentin surface. Peritubular dentin was present (arrow) in round dentinal tubules. The dentinal tubules were approximately 6–8 µm wide.

In the CHX-Ketac™ group, the dentin was applied with 2% CHX for 1 min and restored with H-GIC (Ketac Universal™). The intertubular dentin was flat and was the widest among the 4 groups (Fig. 4H). Many single and clustered particles were scattered on the dentin surface (arrow). Peritubular dentin was evident in all dentinal tubules. The dentinal tubules orifices in this group had the smallest diameter. The tubules were approximately 2–4 µm wide.

## Discussion

The present study evaluated the remineralization effect of CHX used as a cavity disinfectant on dentin carious lesions restored with H-GIC using micro-CT. Our results demonstrated that applying CHX disinfectant prior to restoration improved the MMD gain in the CHX-Equia^TM^ and CHX-Ketac^TM^ groups compared with the non-CHX–treated groups. A previous study, found that cavity conditioner partially demineralized dentin and caused microporosities [41]. Therefore, CHX might neutralize the dentin surface before it is applied with acid conditioner [33].

Sealing carious lesions and bacteria after selective removal and ART is based on the concept of changing the ecological environment by depriving the bacteria of nutrition and reducing or inhibiting bacterial proliferation and activity [42, 43]. To increase the antibacterial effect of sealing by reducing the remaining bacteria, several studies have investigated using an antibacterial agent as a cavity disinfectant or restorative material [2, 29, 42–44]. CHX is a well-known antimicrobial agent used as a cavity disinfectant in ART [27]. CHX is commonly used in dentistry, such as oral surgery, endodontics, prevention, and prophylaxis. CHX is used in different formulations, such as mouthwashes, gels, galenic preparations, solutions, creams, or dentifrices [29]. CHX solutions are typically used in ART. CHX disrupts the cell membrane leading to cell death. A 2% CHX solution killed oral microbes, such as *S. aureas*, *E. faecalis*, *C. albicans*, *P. endodontalis*, *P. gingivalis*, and *P. intermedia* within 60 s [45].

The effect of CHX on promoting remineralization might be explained via two mechanisms. CHX inhibits two collagen-degrading enzymes present in dentin, matrix metalloproteinases (MMPs) and cysteine cathepsins [46, 47]. The MMPs are inactive when the dentin matrix structure is mineralized [48]. Acid production from cariogenic bacteria or acid etching demineralizes the dentin, which activates matrix metalloproteinases and cathepsins that degrade the dentin [36]. The exposed collagen network after acid etching can be degraded by endogenous metalloproteinases, which results in the degradation of the adhesive/dentin interface [49–51]. CHX has been shown to prevent the cross-linked collagen in dentin from degrading by inhibiting matrix metalloproteinase (MMP) activity through a cation-chelating mechanism [37, 51, 52]. The inhibitory effect of CHX on MMPs tended to be dose-dependent and remained active at low concentration 6 months after application [52].

Dentin remineralization is encouraged from the remaining scaffold collagen fibrils that contain minerals [37, 53]. Extra-fibrillar mineral, intra-fibrillar mineral are inorganic structures surrounding the collagen fibrils. Intra-fibrillar mineral affects the elastic behavior in collagen fibrils and resist demineralization. Intra- and extra-fibrillar mineral regrowth in partially demineralized dentin should allow for the recovery of its mechanical properties. Reincorporating collagen fibrils with mineral promotes remineralization [54].

A meta regression study also found that the effect of CHX on inhibiting MMPs might depend on the adhesive system used [55]. For resin restorations, the sequential application of phosphoric acid, CHX, and an etch-and-rinse adhesive may more effectively inhibit MMP activity than the self-etching adhesives because CHX acts better on exposed collagen fibrils [55]. This observation corresponds with our results that CHX had a greater remineralization effect in the Equia™ group, which had a dentin conditioner step that contains a mild polyacrylic acid that can expose the collagen fibrils similar to etch-and-rinse adhesive systems. In contrast, Ketac™ does not require a dentin conditioner step; therefore, the dentin is not demineralized and the MMP inhibitor effect of CHX does not occur. This likely explains why no significant difference in MMD gain was observed between the Ketac^TM^ and CHX-Ketac^TM^ groups.

Another possible mechanism by which CHX might promote remineralization is via electrostatic attraction [37]. The interaction between CHX and its target results from a cationic-anionic reaction. The cationic part of the CHX molecule can bind to the negatively charged area of the target substrate. CHX bound to dentin collagen might strongly attract the negatively charged phosphate from hydroxyapatite and H-GIC via the electrostatic interaction between the protonated amine groups of CHX and the mineral phosphates that promote mineral growth and deposition in demineralized dentin [56, 57].

The Ketac™ and CHX-Ketac™ group were not treated with dentin conditioner, thus, dentin demineralization by the mild acid conditioner did not occur in these groups. The collagen fibrils were undegraded and maintained for remineralization as observed in the micrographs of the Ketac™ and CHX-Ketac™ groups. In the Ketac™ group, the dentinal tubule orifices were usually visible and rounded. Peritubular dentin was present on the dentinal tubule orifices. Among the 4 groups, the CHX-Ketac™ group exhibited the thickest intertubular dentin with the smallest dentinal tubule diameters indicating mineral deposition [37]. Our SEM results corresponded with the micro-CT results where the MMD gain post-restoration was highest in the CHX-Ketac™ group.

In the Equia™ group, the intertubular and peritubular dentin were removed by the dentin conditioner. The SEM image of this group demonstrated an irregular eroded surface with very little peritubular dentin that had been highly demineralized. In contrast, the CHX-Equia™ group had a flat surface with peritubular dentin typically seen in the tubules. Interestingly, the intertubular dentin was thicker compared with the Equia™ group. Moreover, precipitates were present on the dentin surface and peritubular dentin in the dentinal tubules. Our results were in accordance with previous studies that found a dense granular deposition of nanoparticles after applying CHX [37, 58]. Based on the SEM images and the micro-CT results, the dentin conditioner demineralized dentin, while the CHX neutralized the action of the dentin conditioner.

To date, our study is the first report to quantify the remineralization effect of CHX on demineralized dentin post-restoration with H-GIC using micro-CT analysis. In this study, we chose to analyze the remineralization in actual dentin carious lesions to mimic the clinical scenario as much as possible. However, the naturally occurring cavities were different in size, shape, and baseline mineral content, which may influence the remineralization quantity at the H-GIC-dentin interface. A large multi-surface cavity with a large H-GIC contact area might exhibit more mineral gain compared with a small cavity. Furthermore, the chemical pH cycling model used to imitate the oral environment [40] had a minimal effect in our study. This might be because most of the cavities were deep class I cavities. The pH cycling solution could not reach bottom of the restored cavities, therefore no significant differences in MMD were observed in any group post-pH cycling. Moreover, the slight increase in MMD post-pH cycling might be due to the exchange of charged ions between the H-GIC and tooth structure.

Our results corresponded with a previous study that investigated the remineralization of dentin as shown by elastic modulus [37]. This study found that the elastic modulus of the demineralized dentin block in the CHX-treated group was higher compared with the non-CHX group. Moreover, the higher the concentration of CHX, the higher an elastic modulus was found. Therefore, applying CHX on demineralized dentin is effective in promoting the remineralization of deep residual caries.

Although using CHX could be beneficial in the ART method due to its antimicrobial and remineralization effect, the effect of CHX on GI bond strength is unclear. Recent studies found that there was no significant difference in bond H-GIC bond strength after applying CHX [33–35]. Given that new H-GICs are being developed, it will be beneficial to see how CHX affects the bond strength and stability. Moreover, the antimicrobial effect of CHX might reduce pulpal pathology from developing in deep dentin caries cases post-restoration. Thus, clinical studies on the survival rate of ART-treated teeth when using CHX with an H-GIC restoration are of particular interest to improve the clinical success of ART in the future.

## Conclusion

Our results indicated that the groups that used 2% CHX as a cavity disinfectant with H-GIC restoration had a higher mean mineral density gain compared with the groups with H-GIC restoration alone. When the dentin was demineralized, CHX increased remineralization by neutralizing the acid effects of the dentin conditioner, maintaining the collagen fibrils, and mineral phosphate attraction. Thus, 2% CHX enhances the remineralization of the dentin adjacent to the H-GIC restoration. Using CHX as a cavity disinfectant is beneficial to ART due to its antimicrobial and remineralization effects. The limitation of this study is that it was an in vitro study. Our results need to be confirmed by clinical studies of the remineralizing effects of CHX used as a cavity disinfectant. Furthermore, clinical studies on the survival rate of ART-treated teeth when using CHX with an H-GIC restoration are of particular interest to improve the clinical success of ART in the future.

## Data Availability

The datasets generated and/or analyzed during the current study are not publicly available due to Chulalongkorn University thesis copyright but are available from the corresponding author on reasonable request.

## Abbreviations

ART: Atraumatic restorative treatment
CHX: Chlorhexidine gluconate
°C: Celsius
gr: Gram
H-GIC: High viscosity glass ionomer cement
h: Hours
kVp: Kilovoltage peak
MMD: Mean mineral density
Micro-CT: Micro-computed topography
min: Minute
mm: Millimeter
mm^2^: Square millimeter
mm^3^: Cubic millimeter
mg/cm^3^: Milligram per cubic centimeter
mM: Millimolar
μA: Microampere
μm: Micrometer
psi: Pound per square inch
sec: Second
2D: Two-dimensional
3D: Three-dimensional
